# Analysis of DNA Methylation Profiles in Mandibular Condyle of Chicks With Crossed Beaks Using Whole-Genome Bisulfite Sequencing

**DOI:** 10.3389/fgene.2021.680115

**Published:** 2021-07-08

**Authors:** Lei Shi, Hao Bai, Yunlei Li, Jingwei Yuan, Panlin Wang, Yuanmei Wang, Aixin Ni, Linlin Jiang, Pingzhuang Ge, Shixiong Bian, Yunhe Zong, Adamu Mani Isa, Hailai Hagos Tesfay, Fujian Yang, Hui Ma, Yanyan Sun, Jilan Chen

**Affiliations:** ^1^Institute of Animal Sciences, Chinese Academy of Agricultural Sciences, Beijing, China; ^2^Joint International Research Laboratory of Agriculture and Agri-Product Safety, The Ministry of Education of China, Institutes of Agricultural Science and Technology Development, Yangzhou University, Yangzhou, China; ^3^Guangxi Shenhuang Group Co., Ltd., Yulin, China

**Keywords:** chicken, crossed beak, epigenetics, DNA methylation, integration analysis, *FIGNL1*

## Abstract

Crossed beaks have been observed in at least 12 chicken strains around the world, which severely impairs their growth and welfare. To explore the intrinsic factor causing crossed beaks, this study measured the length of bilateral mandibular ramus of affected birds, and investigated the genome-wide DNA methylation profiles of normal and affected sides of mandibular condyle. Results showed that the trait was caused by impaired development of unilateral mandibular ramus, which is extended through calcification of mandibular condyle. The methylation levels in the CG contexts were higher than that of CHG and CHH, with the highest methylation level of gene body region, followed by transcription termination sites and downstream. Subsequently, we identified 1,568 differentially methylated regions and 1,317 differentially methylated genes in CG contexts. Functional annotation analysis of Gene Ontology and Kyoto Encyclopedia of Genes and Genomes showed that these genes were involved in bone mineralization and bone morphogenesis. Furthermore, by combining the WGBS and previous RNA-Seq data, 11 overlapped genes were regulated by both long non-coding RNA and DNA methylation. Among them, *FIGNL1* is an important gene in calcification of mandibular condyle. Generally, because the affected genes play key roles in maintaining mandibular calcification, these changes may be pivotal factors of crossed beaks.

## Introduction

The beak consists of the maxillary and mandible, which are the main facial feature of birds (Gottschaldt and Lausmann, 1974). Crossed beak is a deformity defined earlier as misalignment of the upper and lower beak (Pomeroy, 1962), and the prevalence ranging from 0.2 to 7.4% was documented in at least 12 chicken strains around the world (Landauer, 1938, 1956; Joller et al., 2018; Hong et al., 2019; Shi et al., 2020). In addition, this trait exists in about 30% of 114 Chinese native chicken strains, according to our survey (Shi et al., 2020). Our previous study also showed that crossed beaks are frequently presented after hatch, and the crossed angle had been more and more serious with age until 56 days (Shi et al., 2020). Generally, chicks with crossed beaks have reduced feed intake (Benkman and Lindholm, 1991), inhibited growth (Chen et al., 2011; Giuseppe et al., 2015), poor performance (Hong et al., 2019), and shorter survival (Bai et al., 2018a, b; Joller et al., 2018), which is a great problem for the birds.

Crossed beak is a complex trait regulated by many genes, and its heritability was estimated to be 0.1 (Bai et al., 2018a). The genetic determinants of the complex trait have been studied at the genomic (Bai et al., 2018a, b; Joller et al., 2018), transcriptional (Bai et al., 2014), and translational levels (Sun et al., 2019). However, the genetic determinants of crossed beaks remain incompletely understood. DNA methylation is an epigenetic regulatory mechanism, which mediates numerous biological processes, such as growth, development and genomic imprinting (Smith and Meissner, 2013). Li C. W. et al. (2015) collected embryos and post-hatched chicks to study the level of global DNA methylation, and found that spatiotemporal specific epigenetic alterations could be critical for the late development of chick embryos and neonates. Xiao et al. (2016) evaluated DNA methylation in mandibular head cartilage in rat, and identified that 440 consistently changed genes in early, middle, and late phases of temporomandibular joint osteoarthritis, and 80% of which were hypomethylated and related to cell cycle regulation. In addition, the genome-wide methylation profile of bone revealed differentially methylated regions (DMRs) in osteoporosis and osteoarthritis, which enriched in genes associated with cell differentiation and skeletal embryogenesis (Delgado-Calle et al., 2013). These studies indicate that DNA methylation plays an important role in bone development. However, little is known about the expression patterns and potential roles of DNA methylation in beak development, especially in the complex genetic disease of crossed beak.

A previous study has shown that crossed beaks from 14 to 70 days of age were characterized with impaired development of unilateral mandibular ramus, and mandibular condyle is the growth center for the mandibular ramus extension (Shi et al., 2020). Moreover, Fukaya et al. (2017) discovered that unilateral IGF-1 injection extensively up-regulated the genes including *RUNX2*, *COL2*, and *IHH* in the mandibular condyle, and induced endochondral growth and a lateral shift of the mandible to the response side. This observation identifies the expression of molecular asymmetry may determines morphological left-right asymmetry in beaks. Based on above, we suspect that the epigenetic regulatory mechanisms of bilateral mandibular condyles of crossed beak chicks may be different, leading to asymmetric calcification of mandible. Therefore, this study systematically analyzed the DNA methylation profiles on both side mandibular condyle of crossed beak chicks using whole-genome bisulfite sequencing (WGBS) technology, aiming to provide new insights into the genetic basis of crossed beaks.

## Results

### Mandibular Length and Body Weight of Normal and Affected Birds

**FIGURE 1:**
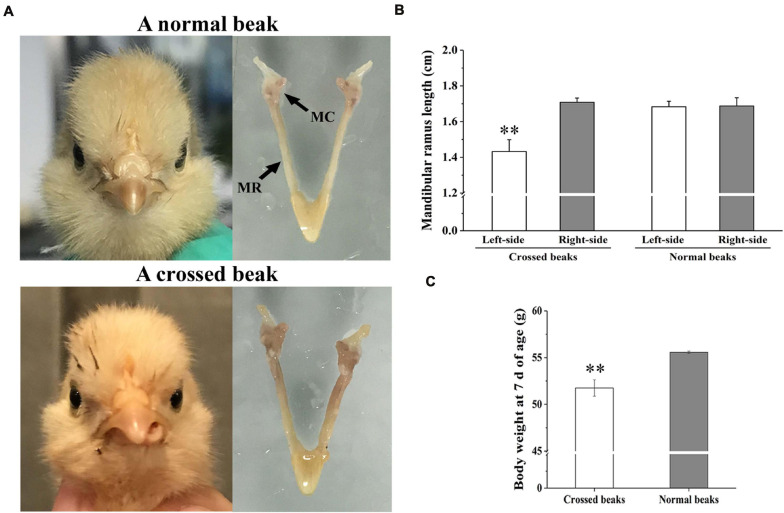
Morphology, mandibular ramus length, and body weight of chicks at 7 days of age. **(A)** Morphological observation of mandible in a normal beak chick and a crossed beak chick (Shi et al., 2020) with left mandibular curvature. MC means mandibular condyle; MR means mandibular ramus. **(B)** The length of bilateral mandibular ramus of crossed beaks with left mandibular curvature (*n* = 16) and normal beaks (*n* = 16). Results are expressed as means ± standard deviation, means were compared by Student-Newman-Keuls multiple-range tests. **(C)** Body weight of 7 day-old chicks with crossed beaks (*n* = 16) and normal beaks (*n* = 16). The body weight was analyzed using *t*-test, ** represents *p* < 0.01.

The affected birds of 7 days of age was caused by asymmetric length of bilateral mandibular ramus (Figure 1A). In particular, the left-side mandibular ramus with left mandibular curvature was shorter than the right-side (*P* < 0.01; Figure 1B). However, there was no difference among the normal right-side ramus of affected birds and two side ramus of normal birds. Meanwhile, the body weight of affected birds was lower than those of normal ones (*P* < 0.01; Figure 1C), which indicated that the beak deformity significantly decreased the growth.

### Genome-Wide DNA Methylation Profiling

The short left-side mandibular condyle of each four affected chicks were mixed in one composite sample and denoted as L group, their corresponding normal right-side condyle was mixed as R group. There are four replicates for each group in total. Global DNA methylation analysis of the four replicates was performed by WGBS with 35 × genome coverage and >99% conversion efficiency. A total of averagely 35.67, and 35.76 clean base were generated for the affected left-side condyle (L) and the normal right-side condyle (R), respectively. After filtering out low-quality data, approximately 71.43 million clean reads with Q30 ranging from 91.40 to 91.92% were generated for each replicate (Table 1). By aligning to chicken genome, the mapped reads were used for subsequent analysis with mapping rate ranging from 81.61 to 82.70%. Detailed quality of sequencing data is shown in Table 1.

**TABLE 1:** Sequencing data by whole genome bisulfite sequencing (WGBS) for left-side (affected-side; L) and right-side (normal-side; R) mandibular condyle of crossed beak chicks with left mandibular curvature.

Group	Composite sample	Clean base (Gb)	Clean reads	GC (%)	Q30	Mapped (%)	Bisulfite conversion rare (%)	Total methylated cytosine (%)
L	L1	35.56	118,527,442	22.00	91.64	82.35	99.43	4.33
	L2	35.37	118,905,521	22.08	91.56	82.34	99.41	4.34
	L3	35.94	119,794,946	21.97	91.59	82.67	99.42	4.33
	L4	35.81	119,352,937	21.96	91.40	81.61	99.28	4.29
R	R1	36.19	120,643,823	21.95	91.70	82.56	99.44	4.37
	R2	35.72	119,080,003	21.92	91.92	82.70	99.39	4.32
	R3	35.56	118,531,096	22.05	91.57	82.50	99.43	4.34
	R4	35.58	118,596,170	21.96	91.75	82.59	99.32	4.30

All methylated genomic C sites were approximately 4.33% (Table 1). The methylation level of CG, CHH, and CHG (where H is A, C, or T) was significant different. In L group, genome-wide methylated cytosine (mC) levels were 89.66, 1.69, and 8.66% for CG, CHG, and CHH, respectively, and those of R group were 89.56, 1.71, and 8.74% (Supplementary Figure 1).

A violin graph was drawn with points representing different methylation levels. The CG methylation levels were high with wide sections in the violin graph, while CHG and CHH methylation levels were low with narrow sections (Supplementary Figure 2). Chromosome methylation maps for all composite samples were plotted (Supplementary Figure 3). The results showed that most chromosomal cytosine hypermethylation was found in the CG context.

We took the 3,000 bp upstream of a gene as promoter region, made an overlap annotation on CpG islands with methylation levels >0.7 and mC coverage > 5×, but not including the hypermethylated CpG island with C-degree confidence less than 0.1. In two groups, hypermethylated CpG islands were found in the distal intergenic regions (Supplementary Figure 4).

**FIGURE 2:**
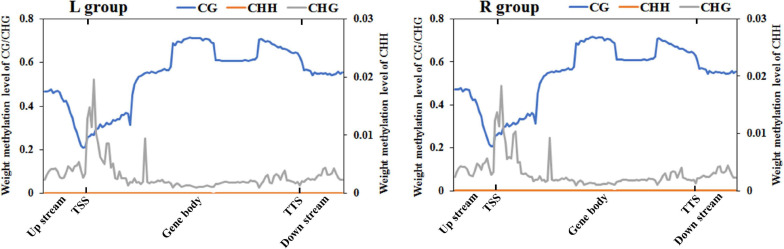
DNA methylation levels across genomic elements in the left-side (affected-side; L) and the right-side (normal-side; R) mandibular condyle of crossed beak chicks with left mandibular curvature. TSS and TTS represents the transcription start site and transcription termination sites, respectively. The blue, origin, and gray solid lines represent CG, CHH, and CHG, respectively.

To further compare the genome-wide distribution and the methylation levels of various functional genomic elements, the methylation status of three different regions were analyzed, including upstream, gene body, and downstream regions (Figure 2 and Supplementary Table 1). In the two groups, there was no significant difference among different genetic elements of the three mC contexts. However, the methylation levels in the CG context were higher than those in the CHG and CHH contexts, where the CHH context was hypomethylated except for the transcription start site (TSS), while CHG context was almost completely unmethylated. The DNA methylation levels in the CG context were the highest in gene body region, then followed by transcription termination sites (TTS) and downstream regions, with sites near the TSS showing the lowest level. The methylation levels gradually decreased from the upstream to the TSS and increased from the TSS to the gene body region. In contrast, the DNA methylation levels in the CHH context decreased from the TSS to the gene body region.

### Characterization of DMRs

**FIGURE 3:**
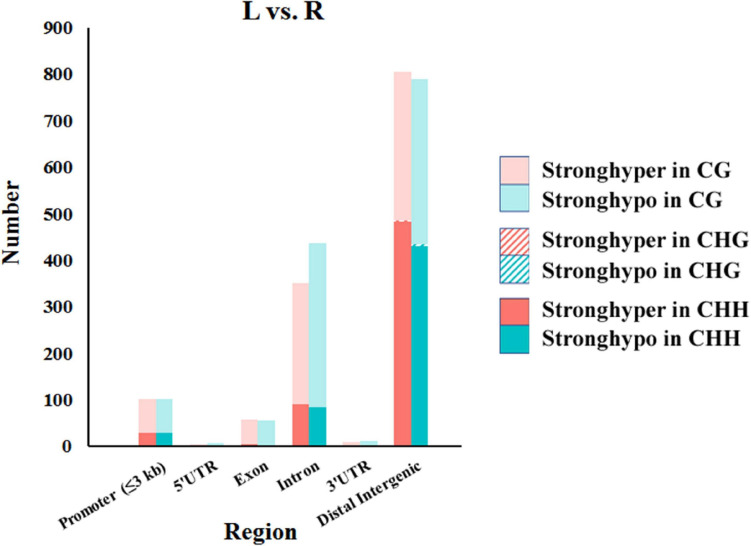
Number of differentially methylated regions (DMRs) in different genomic elements among the left-side (affected-side; L) and the right-side (normal-side; R) mandibular condyle of crossed beak chicks with left mandibular curvature.

**FIGURE 4:**
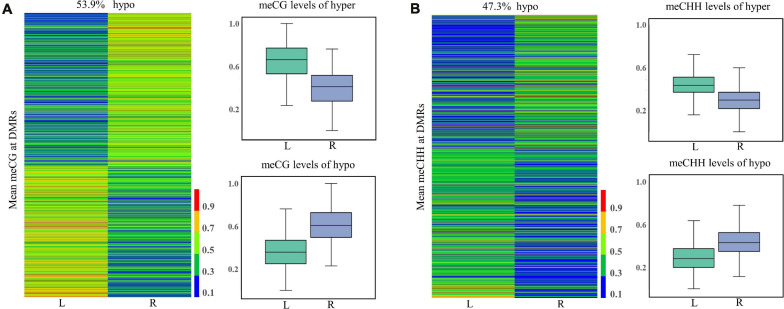
Differentially methylated regions (DMR) dynamics in the left-side (affected-side; L) and the right-side (normal-side; R) mandibular condyle of crossed beaks. Heatmap of DNA methalation profiles and boxplot showing DNA methylation value distribution of DMRs in CG **(A)** and CHH **(B)** contexts.

In total, 1,568, 7, and 1,153 DMRs were identified in CG, CHG, and CHH contexts, respectively. As compared to the normal side, 1,330 (721 CG, 3 CHG, and 606 CHH) were hypermethylated and 1,398 (847 CG, 4 CHG, and 547 CHH) hypomethylated in the affected side. The DMRs were mostly located at distal intergenic regions, followed by introns, regulatory regions, and exons (Figure 3). In the CG context, only 145, 10, and 21 DMRs were in promoters, 5′UTRs, and 3′UTRs, respectively. In addition, as shown in the heat maps in Figure 4, the results showed a clear separation between the left-side and right-side mandibular condyle of crossed beak chicks. Formation of 53.9 and 47.3% hypomethylated DMRs in CG and CHH contexts, respectively. More detailed information is listed in Supplementary Table 2.

### Gene Ontology (GO) and Kyoto Encyclopedia of Genes and Genomes (KEGG) Enrichment Analysis of Differentially Methylated Genes (DMGs)

**FIGURE 5:**
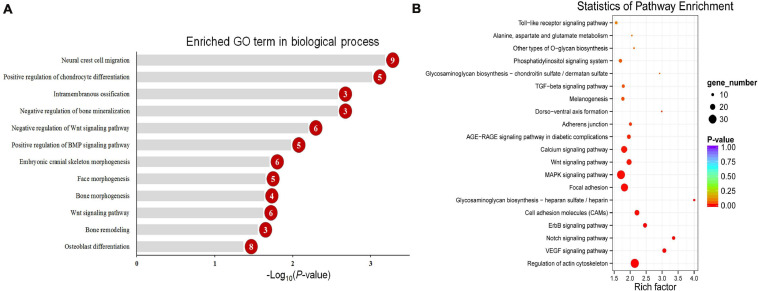
The enriched GO term in biological process related with calcification **(A)** and top 20 enriched KEGG pathways **(B)** of CG context differentially methylated genes (DMGs). Numbers in the red circles represent the gene number.

**FIGURE 6:**
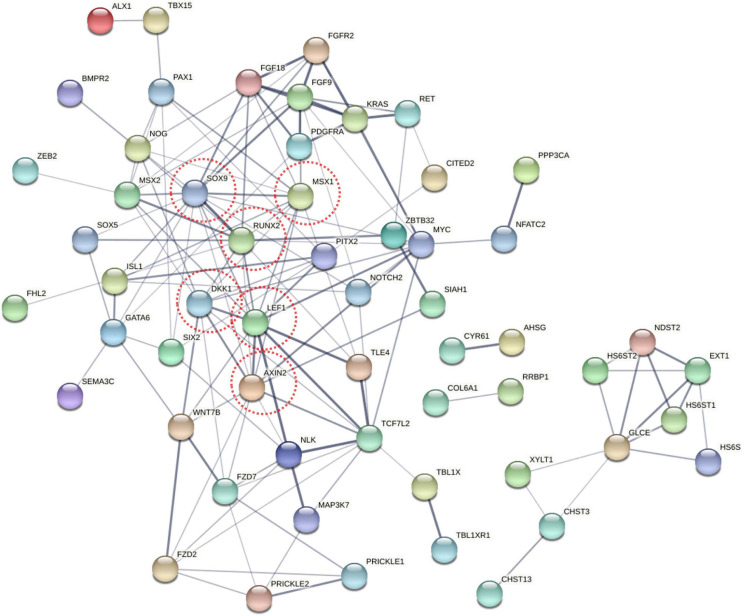
The network of 76 different methylated genes (DMGs) from KEGG pathway and GO terms on CG context. Analysis of the interaction uses STRING software according to the interplay index (confidence >0.4). The interplay index between genes was represented by edge width and transparency. Dark and wide edges indicated high confidence.

To explore the change in the methylation status of genes, the GO and KEGG databases were used to annotate (Supplementary Table 3). Because most of the DMGs is CG and CHH methylation context, we focused on CG and CHH methylation for the DMG functional enrichment analysis. The GO enrichment analysis indicated that the 1,317 DMGs in CG methylation were significantly enriched in negative regulation of bone mineralization, bone morphogenesis, osteoblast differentiation, and so on (Figure 5A and Supplementary Table 4). In detail, there were three genes from negative regulation of bone mineralization, i.e., *SOX9*, *AHSG*, and *BCOR*; four genes from bone morphogenesis, i.e., *PAX1*, *MSX1*, *PAPPA2*, and *CITED2*, and eight genes from osteoblast differentiation, i.e., *FIGNL1*, *FZF9*, and so on. Most of the potential target genes were enriched in KEGG pathways of glycosaminoglycan biosynthesis-chondroitin sulfate/dermatan sulfate, glycosaminoglycan biosynthesis-heparan sulfate/heparin and Wnt signaling pathway (Figure 5B and Supplementary Table 5). The DMGs involved in the three pathways are *CHST3*, *CHST13*, *NDST2*, *GLCE*, *DKK1*, *PRICKLE1*, *LEF1*, *AXIN2*, *MAP3K7*, *LOC101748851*, and so on. In addition, the interaction network of 76 DMGs from three pathways that mentioned above and all GO terms was generated using STRING software. As shown in Figure 6, *SOX9*, *RUNX2*, *MSX1*, *AXIN2*, *DKK1*, and *LEF1* were identified as hub genes in the interaction network related to the calcification of mandibular condyle.

**FIGURE 7:**
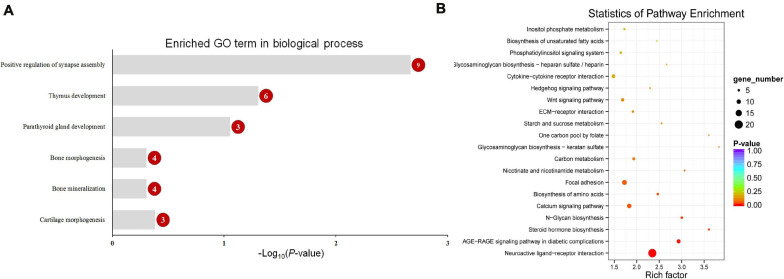
The enriched GO term in biological process related with calcification **(A)** and top 20 enriched KEGG pathways **(B)** of CHH context differentially methylated genes (DMGs). Numbers in the red circles represent the gene number.

Functional enrichment analysis showed that 660 DMGs in CHH methylation were significantly enriched in neural crest cell migration, bone morphogenesis, and so on (Figure 7). More detailed results of the GO and KEGG analyses in CG and CHH methylation are shown in Supplementary Tables 6, 7.

### Expression Pattern of FIGNL1, MSX1, RUNX2, SOX9, CHST3, and CHST13

**FIGURE 8:**
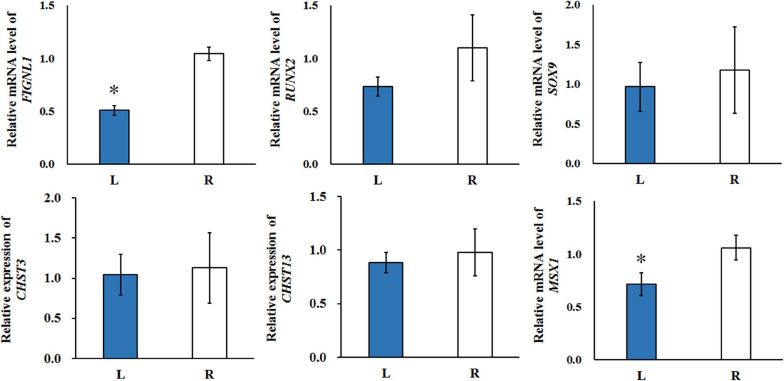
The qRT-PCR was performed to detect the relative mRNA expressions in the left-side (affected-side; L) and the right-side (normal-side; R) mandibular condyle of crossed beak chicks of 7 days-old age with left mandibular curvature. *GAPDH* was used as internal control. Values are expressed as means ± standard deviation of four replicates. The expression data were analyzed using *t*-test, ^∗^ represents *p* < 0.05.

The expression of potential genes related with calcification were evaluated using *T*-test (Figure 8). The quantitative real-time polymerase chain reaction (qRT-PCR) analysis showed that the expression of *FIGNL1* and *MSX1* in affected-side mandibular condyle were lower than that of normal-side (*P* < 0.05). However, the expression of *RUNX2* and *SOX9* were not significantly different between the two sides mandibular condyle of crossed beaks, but the trend showed that those of 0.67 and 0.82 times, respectively lower in the affected-side than the normal-side. Moreover, there was no difference in expression of *CHST3* and *CHST13* (*P* > 0.05).

### Integration Analysis of DMGs, Differentially Expressed Genes (DEGs), and Differentially Expressed Long Non-coding RNAs (DE lncRNAs)

A total of 14 genes were identified as both DEGs and DMGs, using our previous RNA-seq data, which similarly compared between the affected left-side and the normal right-side mandibular condyle of affected chicks with left mandibular curvature (Supplementary Table 8), and 11 candidate genes of them were regulated by both lncRNA and DNA methylation. Moreover, most genes were associated with more than one lncRNA, with methylation differences mainly distributed in the distal intergenic and intron regions. More detailed results on the above genes are listed in Table 2.

**TABLE 2:** The 11 candidate genes regulated by both long non-coding RNA (lncRNA) and DNA methylation in the left-side (affected-side; L) and the right-side (normal-side; R) mandibular condyle of crossed beak chicks with left mandibular curvature.

lncRNA	Regulation*	Gene name	L vs. R log_2_FC	Methylation chromosome	Methylation Difference (L vs. R)^†^	*P*-value	DMR	Methtype
MSTRG.25596.1	Trans	DACT1	0.6	5 (55293353–55,293,455)	–0.308	3.60E-10	Exon	CG
MSTRG.36872.1, MSTRG.37605.1, MSTRG.46124.1	Trans	FAM72A	–0.7	26 (2379459–2,379,604)	0.287	4.20E-06	Distal intergenic	CG
MSTRG.22262.17, MSTRG.22262.19	Cis	FIGNL1	–0.64	2 (80755849–80,755,879)	0.276	6.20E-05	Promoter (2–3 kb)	CG
MSTRG.4939.1	Trans	IFI6	–0.83	2 (89711278–89,711,362)	0.241	8.10E-05	Distal intergenic	CG
MSTRG.59623.1, MSTRG.84282.134	Trans	MAP2	0.84	7 (2494584–2,494,594)	–0.215	2.90E-05	Intron	CG
MSTRG.93293.25	Trans	UROD	–0.61	8 (21296458–21,296,558)	–0.23	5.90E-06	Distal intergenic	CG
MSTRG.32999.1, MSTRG.99022.1	Trans	TREM-B2	–0.89	26 (4800298–4,800,313)	–0.201	4.50E-06	Distal intergenic	CG
				26 (4801253–4,801,312)	–0.341	1.20E-11	Distal intergenic	CG
MSTRG.95299.1, MSTRG.95311.1, MSTRG.95322.1	Cis			26 (4801573–4,801,816)	0.215	4.60E-10	Distal intergenic	CG
				26 (4791777–4,791,848)	0.329	2.70E-50	Distal intergenic	CHH
MSTRG.35324.1, MSTRG.45316.1, MSTRG.56264.1, MSTRG.82308.1, MSTRG.87705.1, MSTRG.93293.20, MSTRG.93293.25, MSTRG.98034.1	Trans	ZNF469	0.65	11 (18285285–18,285,311)	–0.249	5.20E-05	Distal intergenic	CG
				11 (18295768–18,295,880)	0.145	6.40E-06	Distal intergenic	CHH
MSTRG.33196.1, MSTRG.46124.1, MSTRG.84593.4, MSTRG.99022.1	Trans	RAB3C	–0.61	Z (18152598–18,152,641)	–0.111	2.00E-07	Intron	CHH
MSTRG.46337.2, MSTRG.59623.1, MSTRG.62713.20, MSTRG.93293.25	Trans	NPAS3	0.74	5 (35248931–35,248,947)	–0.103	1.00E-07	Distal intergenic	CHH
MSTRG.13508.30, MSTRG.33152.1, MSTRG.46124.1, MSTRG.78620.1, MSTRG.84593.4, MSTRG.99022.1	Trans	ARHGAP15	–0.69	7 (32976536–32,976,622)	0.169	2.9E-17	Intron	CHH

*For trans target genes, we calculated the Pearson correlation coefficient (> 0.9) and significant P–value (< 0.01) for the expression levels of each lncRNA-mRNA pair. For cis target genes, we identified chromosomal co-expressed genes within 100 kbps upstream and downstream of DE lncRNAs. ^†^Methylation difference: the difference in methylation levels between L and R; a positive number means the methylation levels of this region in the L group are higher than those in the R group, and a negative number means that the methylation levels of this region in the L group are lower than those in the R group.

**FIGURE 9:**
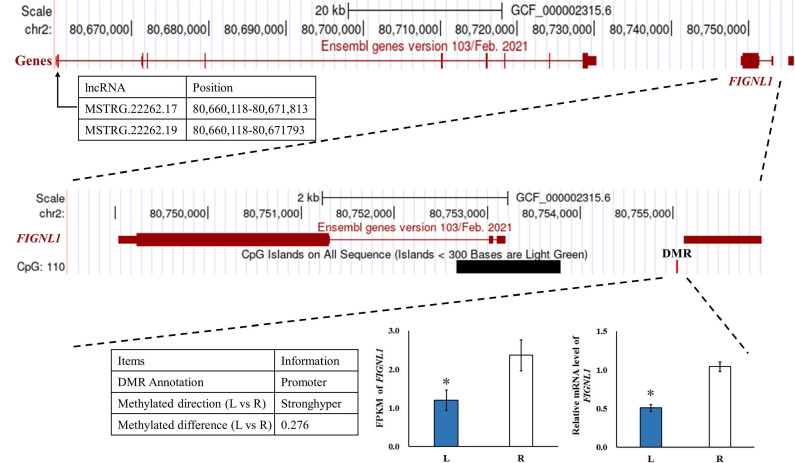
The genome browser track plot around the *FIGNL1* locus. The qRT-PCR was performed to detect the relative mRNA expressions in the left-side (affected-side; L) and the right-side (normal-side; R) mandibular condyle of crossed beak chicks of 7 days-old age with left mandibular curvature. *GAPDH* was used as internal control. Values are expressed as means ± standard deviation of four replicates. The expression data were analyzed using *t*-test, *represents *p* < 0.05.

To investigate the effect of DNA methylation on gene expression levels, we compared the trend between gene expression and methylation levels using the fragments per kilobase million (FPKM) value for the RNA-seq data and the difference in methylation levels between L and R WGBS data samples (Table 2). The results showed that the DNA methylation level in the promoter regions of *FIGNL1* was opposite of that observed for their expression levels. Furthermore, qRT-PCR results showed that the expression levels of the *FIGNL1* was down-regulated in the affected left-side mandibular condyle of crossed beak chicks (*P* < 0.01; Figure 9).

## Discussion

Within recent years, the sporadic occurrence of crossed beaks has been described by researchers in wildbirds (van Hemert et al., 2012; van Hemert and Handel, 2016) as well as in chickens (Handel et al., 2010; Joller et al., 2018; Hong et al., 2019). These affected chickens are usually normal at hatch, and do not become apparent until 1–2 months old (Landauer, 1938). Meanwhile, the type of mandible deviating laterally from the longitudinal axis of the head was more frequent (Bai, 2017; Joller et al., 2018; Hong et al., 2019). In this study, bilateral mandibular ramus length of crossed beaks chicks was asymmetrical at 7 day of age, where the affected shorter one is shorter than normal, and the other one is similar to that of the normal chicks. These results are similar to our previous study that crossed beaks had short of unilateral mandibular ramus from 14 to 70 day of age (Shi et al., 2020). In addition, many researches indicated that the condyle is essential for mandibular growth, in particular for the enlargement of the ramus (Meikle, 1973; Pirttiniemi et al., 2009). Thus, we suspect that there may be different epigenetic regulatory mechanisms for the growth of affected-side and normal-side mandibular ramus in a crossed beak, and caused the bone growth differently. Meanwhile, the mandibular condyle as the key point of bone development is an ideal sample for studying molecular mechanism of this complex trait.

DNA methylation is an epigenetic regulation form with important roles in gene expression and tissue development (Zhang et al., 2019). Although bone DNA methylation has been analyzed in human (Wu et al., 2019), baboon femora (Housman et al., 2017), and rat (Villagra et al., 2002; Nagaoka et al., 2019), to our knowledge, this is the first systematic comparison of genome-wide DNA methylation profiles of bilateral mandibular condyle in chickens. In the present study, bilateral mandibular condyles of genome-wide methylation patterns were similar in functional genomic regions. However, there were differences among three mC contexts which might be related to differences in the sequences of different genetic elements (Fang et al., 2017). Approximately 4.3% of cytosine sites were methylated, which is lower than the cortical bones in mice from 6.06 to 6.48% (Wei et al., 2020). The highest proportion of CG methylation in this study was similar to that found in other species (Wei et al., 2020) and tissues (Li C. W. et al., 2015; Li J. X. et al., 2015; Zhang et al., 2018; Tan et al., 2020). Among the gene functional regions, TSSs presented the lowest methylation levels, which was consistent with the results in chickens’ liver (Tan et al., 2020) and blood (Zhang et al., 2018), but inconsistent in cortical bones of mice (Wei et al., 2020).

We compared the trend between gene expression and methylation levels for the RNA-seq data and the difference in methylation levels to determine whether DMGs play a determinative role in calcification of mandibular condyle. The results showed that *FIGNL1* was regulated by both lncRNA and DNA methylation. The DMRs of this gene is located in the promoter region, and the trend of DNA methylation levels is in contrast with its expression. As shown in a previous study, *FIGNL1* is a subfamily member of the ATPases associated with diverse cellular activities protein family, which plays an important role in the inhibition of osteoblast proliferation and the stimulation of osteoblast differentiation (Park et al., 2007). The over-expression of *FIGNL1* could reduce the proliferation of calvarial cells, and enhance the mRNA expression of osteocalcin and alkaline phosphatase. Therefore, *FIGNL1* is highly expressed in the cells of mineralized tissue and plays a critical function in the formation of hard tissue. In contrast, interference with *FIGNL1* significantly increased the proliferation of osteoblasts and decreased the expression of osteocalcin and alkaline phosphatase (Park et al., 2007). At present, there are few studies on epigenetic modifications or chromatin accessibility of *FIGNL1*. Although, the mechanism of crossed beaks has been studied at the genomic, transcriptional, and translational levels, the genetic determinants of crossed beaks remain incompletely understood. The joint analysis neither results in very consistent conclusions, mostly because the fact that the specific samples varies among studies. With the deepening of the research on the crossed beak, we discovered that the crossed beak was caused by the unilateral short of mandibular ramus, which extends through calcification of mandibular condyle (Shi et al., 2020). Based on the above, the mandibular condyle was studied and the *FIGNL1* was identified by integration analysis of RNA-seq and WGBS. The results in this study indicated that, hypermethylation of the promoter regions may inhibit *FIGNL1* expression on affected-side mandibular condyle of crossed beaks, and the expression of *FIGNL1* was regulated by two lncRNAs. Therefore, *FIGNL1* is an important gene in the process of mandibular calcification, and these results provided two candidate lncRNA and methylation marker for a new regulatory mechanism of calcification in mandibular condyle.

In this study, *RUNX2*, *SOX9*, and *MSX1* are the hub genes identified through the interaction network associated with mandibular condylar calcification. *RUNX2* is a transcription factor essential for skeletal development. Osteoblasts are completely absent in *RUNX2* knockout mice, which indicates that *RUNX2* is an essential transcription factor for osteoblast differentiation (Komori et al., 1997). *SOX9* is a master transcription factor that participates in sequential events in chondrogenesis by regulating a series of downstream factors in a stage-specific manner. A previous study indicated that the physiological down-regulation of *SOX9* in hypertrophic chondrocyte is associated with up-regulation of osteoblast-associated genes (Lefebvre, 2019). In transgenic mice expressing *SOX9*, the number of chondrocytes transdifferentiating into osteoblasts was markedly reduced (Lui et al., 2019). Moreover, *SOX9* can also physically interact with *RUNX2* and may thereby delay the master osteogenic actions of this RUNT-domain transcription factor (Zhou et al., 2006). In this study, the methylation of *RUNX2* and *SOX9* in distal intergenic and intron regions, respectively, may be involved in the formation of crossed beaks. Recent advances in techniques to study genome-wide methylation patterns have facilitated the identification of significant DNA methylation in intergenic and genebody regions. It is speculated that methylation within these non-promoter regions regulate alternative promoters, RNA processing, transposable elements such as long and short interspersed elements, and non-coding RNAs (Kulis et al., 2013). Intergenic DNA hypomethylation that results from dysfunctional trans-regulatory pathway (Weinberg et al., 2019). In addition, DNA methylation within intergenic regions is a mechanism regulating microRNAs (Pheiffer et al., 2016) and lncRNAs (Bermejo et al., 2019). The expression of *RUNX2* and *SOX9* were not significantly different between two sides mandibular condyle, however, the trend showed that those of 0.67 and 0.82 times, respectively lower in the affected-side than the normal-side mandibular condyle. Generally, DNA methylation is one of epigenetic mechanism which regulates gene expression. It remains to be further analyzed whether the above genes are regulated by non-coding RNA and histone modification.

*MSX1* and *PAX1* are two DMGs enriched in bone morphogenesis terms. *MSX1* is a homeobox transcriptional factor and involved in limb-pattern formation and craniofacial development, specifically in tooth formation (Satokata and Maas, 1994; Chung et al., 2010; Nassif et al., 2014). Previous studies have reported that the most striking feature of *MSX1* mutation is the inhibition of the mandibular basal convexity and absence of endochondral ossification in the mandibular condyle (Satokata et al., 2000; Orestes-Cardoso et al., 2009). Meanwhile, *MSX1* is an upstream and downstream regulator for the bone morphogenetic protein *BMP2* and *BMP4* signaling pathway, respectively (Maxence et al., 2013), which stimulates trabecular bone metabolism and controls the collagen-based mineralization process (Nassif et al., 2014). In this study, methylation of the *MSX1* in 3′UTRs regions may be related to the down-regulated of *MSX1* in affected-side mandibular condyle of crossed beaks. *PAX1* indirectly promotes the early stages of chondrogenic differentiation (Rodrigo, 2003), and *PAX1*-misexpressing chondrocytes exhibited abnormal cell morphology (Murtaugh et al., 2001; Takimoto et al., 2013). Moreover, Adhikari et al. (2016) and Shaffer et al. (2016) revealed that *PAX1* play roles in craniofacial development or face syndromes. In this study, *PAX1* had the DMR in exon region, which may be responsible for the calcification. Based on the above, these mentioned genes play important roles in calcification of mandibular condyle, and differentiation and regulation of them through DNA methylation might be one of the mechanisms that determine the difference of mandibular ramus length in crossed beak chicks. Nonetheless, the epigenetic mechanisms involved in the regulation of these genes and genetic regions involved in bone morphogenesis require further study.

This study systematically described the genome-wide DNA methylation patterns of mandibular condyle in chicks for the first time. *FIGNL1*, several important DMRs/DMGs, and pathways were emphasized to be related with calcification of mandibular condyle in crossed beaks. The results provide valuable data for further understanding the genetic and epigenetic mechanisms of this malformation.

## Materials and Methods

### Animals and Sample Collection

The study was conducted according to the local ethical guidelines and met the requirement of the institutional animal care and use committee (No. IAS2020-8). As the incidence of crossed beaks did not differ between male and female progeny based on our previous study (Bai, 2017), 32 female Beijing-You chickens including 16 normal beak chicks and 16 affected chicks with left mandibular curvature were used in this study. All birds were incubated contemporally and kept in the same environment without beak-trimming.

As the incidence increased quickly since 7 day of age (Shi et al., 2020), all chicks were weighed at the age of 7 day in this study, and the length of bilateral mandibular ramus were measured from the photo by Digimizer 5.3.4 MedCalc software (Ostend, Belgium). Meanwhile, two sides mandibular condyles of 16 affected chicks were dissected, temporarily frozen in liquid nitrogen and stored at -80°C.

The short left-side condyle of each four affected chicks were mixed in one composite sample and denoted as L group, their corresponding normal right -side condyle was mixed as R group. There are four replicates for each group in total. Then, genomic DNA was isolated from mandibular condyle tissues of each composite sample replicated using the phenol-chloroform method.

### Library Preparation

The DNA concentration and quality were determined by NanoDrop (NanoDrop Technologies, Wilmington, DE, United States) and agarose gel electrophoresis before library construction. Four DNA libraries for L and R groups, respectively were constructed. Equal amounts of genomic DNA (2 μg per sample) were fragmented to 400–500 bp by ultrasonication, followed by adenylation and end-repair. The selected fragments were treated with bisulfite and then amplified by PCR to generate the sequencing libraries.

### WGBS and Identification of DMRs

The library was sequenced using an IlluminaHiSeqTM2500 platform (Biomarker Technologies, Beijing, China), and the peak signal was transformed into sequence data by base calling, following which the raw reads were quality-filtered to obtain the clean reads. First, 3′ adapter sequence were trimmed. Then, reads with >10% unknown bases (N) and those of low quality (more than 50% of bases with a PHRED score ≤5) were removed. The Q30 and GC content were also calculated.

The clean reads were aligned to the chicken genome (GRCg6a) and the bisulfite mapping of methylation sites was performed using Bismark software. The duplicates were reads that aligned with the same region of the genome, and can estimated the sequencing depth and coverage. The bisulfite conversion rate is the percentage of methylated clean reads to the total number of clean reads in the genome. The binomial distribution test for each C site was used to confirm C site methylation by screening conditions for coverage ≥4 × and false discovery rate (FDR) < 0.05.

To identify DMRs between bilateral mandibular condyle of crossed beak chicks, we referred to a previously reported model (Lister et al., 2011) to estimate the methylation level. All C sites with reads coverage more than 10 × were used for DMR analysis performed in MOABS (Sun et al., 2014). DMRs were defined by the presence of at least three methylation sites in the region, and in which the difference in methylation levels was >0.1 for CHG and CHH context, and >0.2 for CG context, and the *P*-value from Fisher’s exact test was < 0.05. We annotate the DMR regions using ChIPseeker, and gene overlapped with at least one DMR was defined as DMG (Wang et al., 2020).

### Function Enrichment Analysis

GO enrichment analysis of DMGs was implemented by the GOseq R packages based on the Wallenius non-central hypergeometric distribution (Young et al., 2010). KOBAS software was used to analyze the significance of DMGs enrichment in the KEGG pathway (Kanehisa et al., 2016). Pathways with a *P*-value < 0.05 were considered to be significantly enriched. The STRING database^1^ was used to analyze interaction networks of DMGs (Franceschini et al., 2012).

### Integration Analysis of DMGs, DEGs, and DE lncRNAs

Many DEGs and DE lncRNAs previously screened between left-side and right-side mandibular condyle of crossed beak chicks with left mandibular curvature using the Illumina platform. For trans target genes, we calculated the Pearson correlation coefficient (>0.9) and significant *P*-value (< 0.01) for the expression levels of each lncRNA-mRNA pair. For cis target genes, we identified chromosomal co-expressed genes within 100 kbps upstream and downstream of DE lncRNAs. Thereafter, the integrated analysis of the DMGs, DEGs (FDR < 0.05, and | log_2_FoldChange| ≥ 1.5), and DE lncRNAs (*P*-value < 0.05, and | log_2_FoldChange| ≥ 1.2) were further integrated analysis.

### Validation by Quantitative Real-Time Polymerase Chain Reaction (qRT-PCR)

Total RNA from bilateral mandibular condyle of affected birds (*n* = 4) with left mandibular curvature were reverse transcribed into cDNA using the PrimeScript RT Reagent Kit (TaKaRa, Dalian, China) following the manufacturer’s instructions. qRT-PCR was performed on the ABI QuantStudio 7 Flex Real-Time Detection System (Life Technologies Holdings Pte Ltd., United States) using KAPA SYBR Fast universal qPCR kit (Kapa Biosystems, Boston, United States). Using *GAPDH* as a reference, relative-expression levels of six genes (*FIGNL1*, *MSX1*, *RUNX2*, *SOX9*, *CHST3*, and *CHST13*) were quantified using the 2^–Δ^ ^Δ^ ^*Ct*^ method (Livak and Schmittgen, 2001). The primer sequences are listed in Table 3.

**TABLE 3:** Specific primers for qRT-PCR.

Gene name	Primer sequence	Product size (bp)
*FIGNL1*	F:5′-GGCCGTGGCCGTGTCA-3′ R:5′-TTGGCACGGTACTCATCAGC-3′	132
*MSX1*	F:5′-GGAACTGTGGCAGAGAAAGG-3′ R:5′-AATGGCCACAGGTTAACAGC-3′	118
*RUNX2*	F:5′-ACTTTGACAATAACTGTCCT-3′ R:5′-GACCCCTACTCTCATACTGG-3′	192
*SOX9*	F:5′-AAGTCGGTGAAGAACGGG-3′ R:5′-GCTGAGCGTCCGTTTTGG-3′	202
*CHST3*	F:5′-GAACCACCTGGGAAGGGATG-3′ R:5′-AGCACCTCCCGAAAATCCTG-3′	183
*CHST13*	F:5′-CTGCAAAACATGGCCGTCTC-3′ R:5′-TGATCGCTCTCATACAGGGC-3′	109
*GAPDH*	F:5′-ATCACAGCCACACAGAAGACG-3′ R:5′-TGACTTTCCCCACAGCCTTA-3′	121

### Statistical Analysis

Data were analyzed by SAS 9.1 (SAS Institute Inc., Cary, NC). Means of mandibular length were compared by Student-Newman-Keuls multiple-range tests when a significant difference was detected. The body weight, and expression data were analyzed using *T*-test. The results are presented as the means ± standard deviation. A *P*-value < 0.05 (^∗^) and *P*-value < 0.01 (^∗∗^) implied a statistically significant difference and highly significant difference, respectively.

## Data Availability Statement

The raw data of the WGBS-Seq have been submitted to NCBI Sequence Read Archive (SRA) under BioProject accession PRJNA707365. The data will first be made available to download here: https://dataview.ncbi.nlm.nih.gov/object/PRJNA707365?reviewer=nsq5r9d92u82pj62u3ckdo7eha.

## Ethics Statement

The animal study was reviewed and approved by the Institute of Animal Sciences, Chinese Academy of Agricultural Sciences (No. IAS2020-8).

## Author Contributions

LS, HB, JC, and YS contributed to conception, design, methodology, formal analysis and drafted and edited the manuscript. LS, YL, and JY analyzed the results. PW, YW, AN, LJ, PG, SB, YZ, AI, HT, and HM contributed to investigation. FY contributed to resources. All authors have read and agreed to the published version of the manuscript.

## Conflict of Interest

FY was employed by the company Guangxi Shenhuang Group Co., Ltd. The remaining authors declare that the research was conducted in the absence of any commercial or financial relationships that could be construed as a potential conflict of interest. The reviewer YD declared a past co-authorship with one of the authors HB. The handling Editor declared a past co-authorship with one of the authors HB.

## Footnotes

1 http://string-db.org/

## Supplementary Material

Supplementary Figure 1 The proportion of DNA methylation types of the left-side (affected-side; L) and the right-side (normal-side; R) mandibular condyle of crossed beak chicks with left mandibular curvature.

Supplementary Figure 2 Violin plot for the overall distribution of methylation levels for different methylation types. **(a)** CG, **(b)** CHG, and **(c)** CHH. H = A, C or T. The L and R groups means left-side (affected-side) and right-side (normal-side) mandibular condyle, respectively, of crossed beaks chicks with left mandibular curvature. The abscissa represents the different samples, the ordinate represents the level of methylation of the samples; the width of each violin represents the density of the point at that methylation level, while the boxplot shows the methylation levels in each violin.

Supplementary Figure 3 Chromosome methylation maps for allcomposite samples.

Supplementary Figure 4 The pie plot of CpG distribution in each genomic region, respectively.

Supplementary Table 1 DNA methylation levels in gene functional elements in the left-side (affected-side; L) and the right-side (normal-side; R) mandibular condyle of crossed beaks.

Supplementary Table 2 Information of DMRs in in the left-side (af-fected-side; L) and the right-side (normal-side; R) mandibular condyle of crossed beaks.

Supplementary Table 3 Information of DMGs in the left-side (affected-side; L) and the right-side (normal-side; R) man-dibular condyle of crossed beaks.

Supplementary Table 4 The list of DMGs enriched GO terms for CG context.

Supplementary Table 5 The list of DMGs enriched top 20 KEGG pathways for CG context.

Supplementary Table 6 The list of DMGs enriched GO terms for CHH context.

Supplementary Table 7 The list of DMGs enriched top 20 KEGG pathways for CHH context.

Supplementary Table 8 The list genes were identified as both DEGs and DMGs in the left-side (affected-side) and the right-side (nor-mal-side) mandibular condyle of crossed beak chicks.
