# Supplementary figures and images for: Analysis of DNA Methylation Profiles in Mandibular Condyle of Chicks With Crossed Beaks Using Whole-Genome Bisulfite Sequencing
Source: Front Genet. 2021 Jul 8;12:680115. doi: 10.3389/fgene.2021.680115 (PMC8298039; doi:10.3389/fgene.2021.680115)

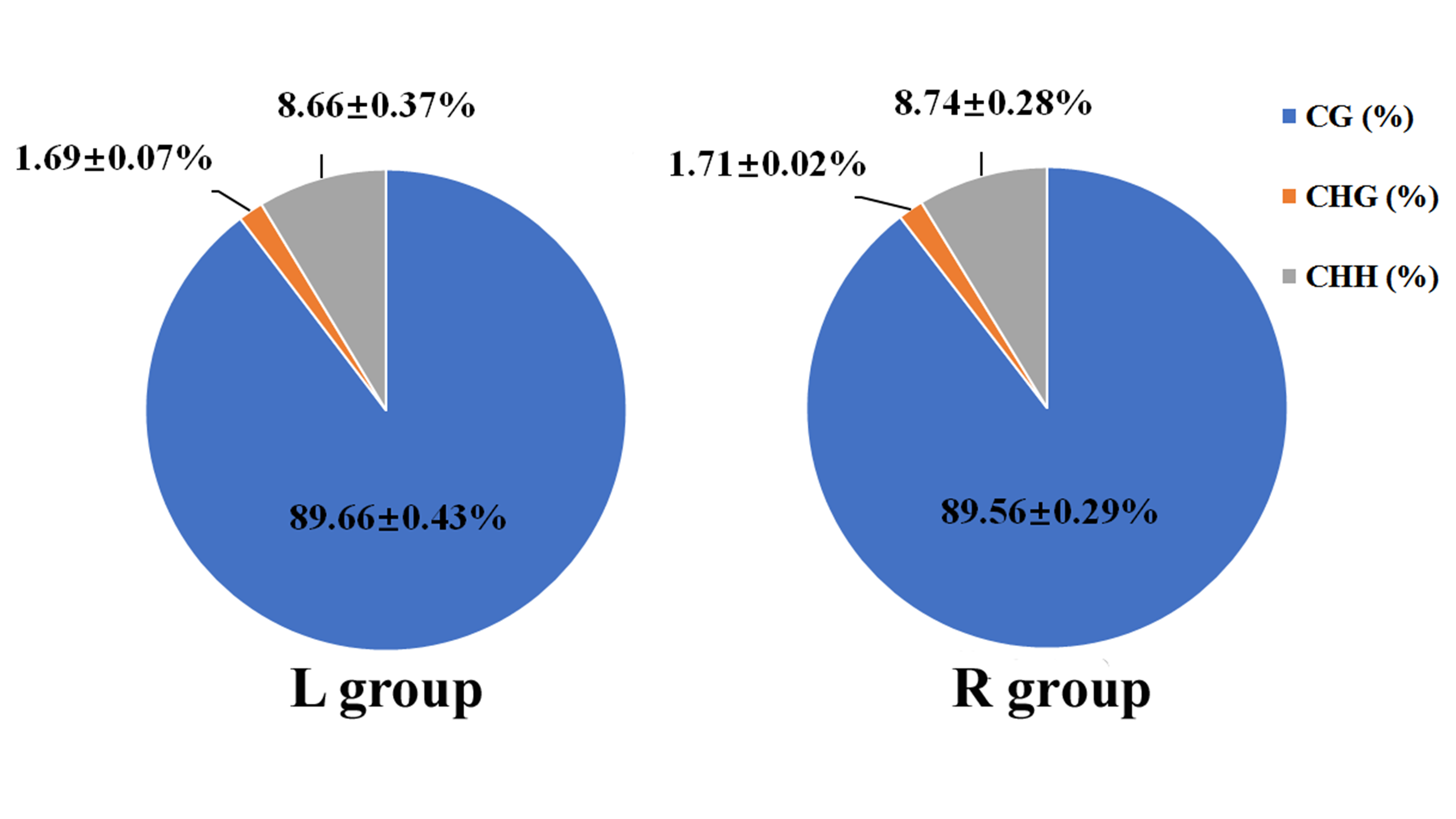

Supplement: Supplementary Figure 1 — The proportion of DNA methylation types of the left-side (affected-side; L) and the right-side (normal-side; R) mandibular condyle of crossed beak chicks with left mandibular curvature. [file Image_1.TIF]

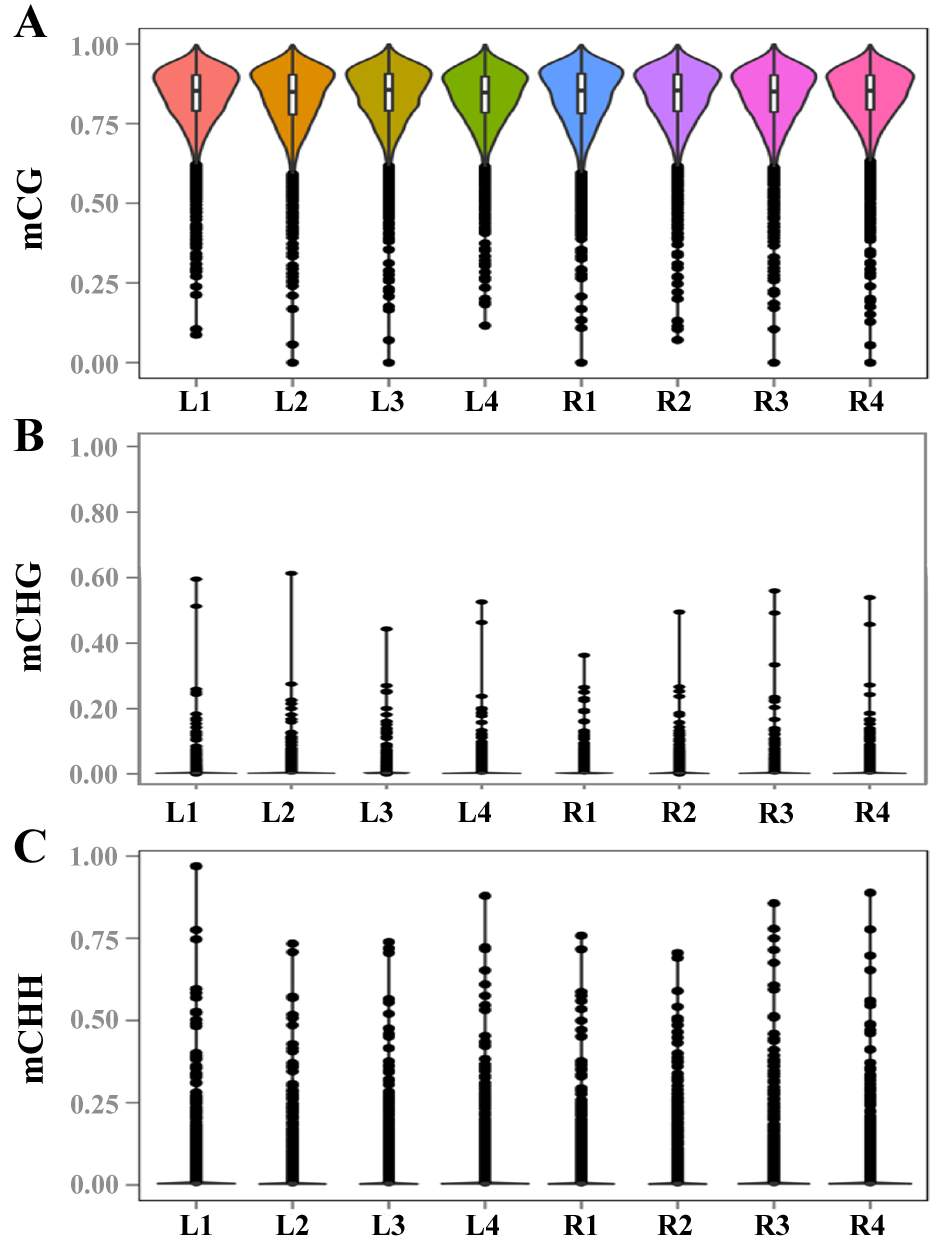

Supplement: Supplementary Figure 2 — Violin plot for the overall distribution of methylation levels for different methylation types. (a) CG, (b) CHG, and (c) CHH. H = A, C or T. The L and R groups means left-side (affected-side) and right-side (normal-side) mandibular condyle, respectively, of crossed beaks chicks with left mandibular curvature. The abscissa represents the different samples, the ordinate represents the level of methylation of the samples; the width of each violin represents the density of the point at that methylation level, while the boxplot shows the methylation levels in each violin. [file Image_2.TIF]

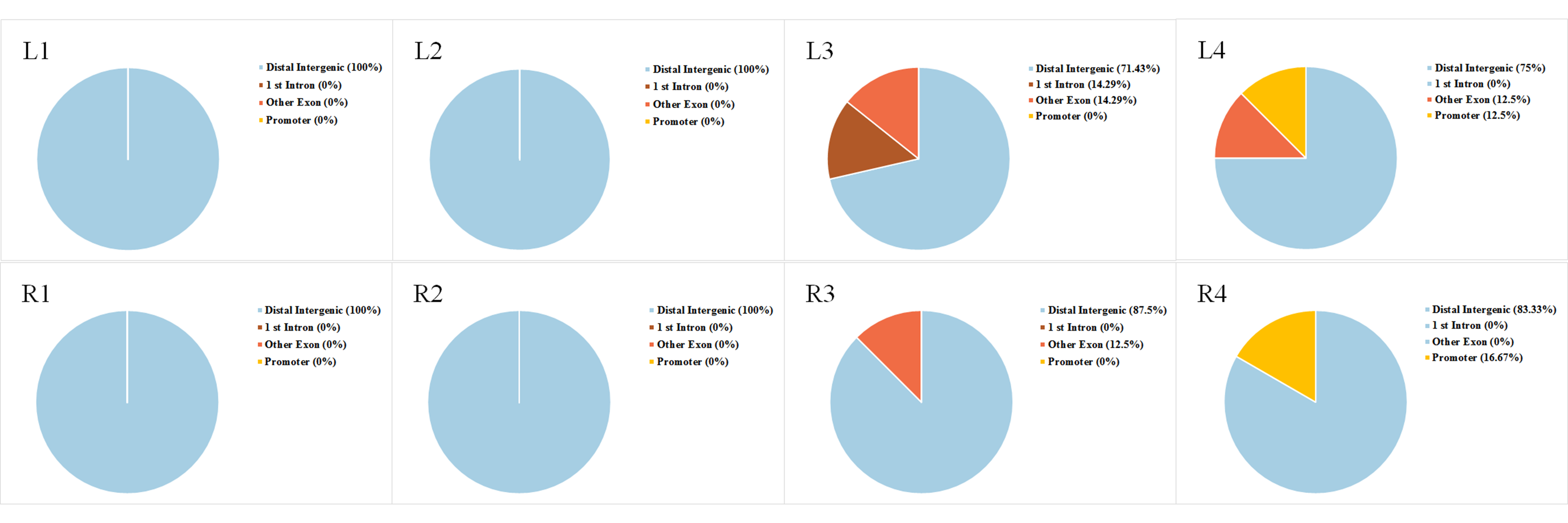

Supplement: Supplementary Figure 4 — The pie plot of CpG distribution in each genomic region, respectively. [file Image_4.TIF]
